# Social Media Discussions Predict Mental Health Consultations on College Campuses

**DOI:** 10.1038/s41598-021-03423-4

**Published:** 2022-01-07

**Authors:** Koustuv Saha, Asra Yousuf, Ryan L. Boyd, James W. Pennebaker, Munmun De Choudhury

**Affiliations:** 1 School of Interactive Computing, Georgia Institute of Technology, Atlanta, GA USA; 2 Department of Psychology, Lancaster University, Lancaster, UK; 3 Security Lancaster, Lancaster University, Lancaster, UK; 4 Data Science Institute, Lancaster University, Lancaster, UK; 5 Department of Psychology, University of Texas at Austin, Austin, TX USA; 6 Present Address: Microsoft Research Lab - Montreal, 6795 Rue Marconi, Suite 400, Montréal, Québec H2S 3J9 Canada

**Keywords:** Health services, Computational science, Computer science, Predictive markers

## Abstract

The mental health of college students is a growing concern, and gauging the mental health needs of college students is difficult to assess in real-time and in scale. To address this gap, researchers and practitioners have encouraged the use of passive technologies. Social media is one such "passive sensor" that has shown potential as a viable "passive sensor" of mental health. However, the construct validity and in-practice reliability of computational assessments of mental health constructs with social media data remain largely unexplored. Towards this goal, we study how assessing the mental health of college students using social media data correspond with ground-truth data of on-campus mental health consultations. For a large U.S. public university, we obtained ground-truth data of on-campus mental health consultations between 2011–2016, and collected 66,000 posts from the university’s Reddit community. We adopted machine learning and natural language methodologies to measure symptomatic mental health expressions of depression, anxiety, stress, suicidal ideation, and psychosis on the social media data. Seasonal auto-regressive integrated moving average (SARIMA) models of forecasting on-campus mental health consultations showed that incorporating social media data led to predictions with r = 0.86 and SMAPE = 13.30, outperforming models without social media data by 41%. Our language analyses revealed that social media discussions during high mental health consultations months consisted of discussions on academics and career, whereas months of low mental health consultations saliently show expressions of positive affect, collective identity, and socialization. This study reveals that social media data can improve our understanding of college students’ mental health, particularly their mental health treatment needs.

## Introduction

Mental health on college campuses is a matter of growing concern as an increasing number of college students show rising levels of anxiety, depression, and suicidal ideation. According to the 2019 National College Health Assessment^1^ 16.7% students felt too depressed to function in the last 2 weeks from when the survey was conducted while 8.6% seriously considered suicide or tried to harm themselves in the past 12 months. Another decade-spanning study found that the percentage of students diagnosed with mental illness rose up from 22% in 2007 to 36% in 2017 even though the rate of treatment increased from 19 to 34%^2^. Mental health services on college campuses, including on-campus counseling centers and psychiatric clinics, therefore continuously struggle to address the increasing demands of mental health consultations in a timely fashion^3^. A research study conducted by Penn State’s Center for Collegiate Mental Health, for instance, reported a 30–40% increase in the on-campus counseling consultations between 2009–2015, despite an only 5% increase in enrollment^4^. In short, these services often lack in resources, staff, and preparedness, leading to long waiting lists and selective/infrequent consultations of many^5^. This underscores an urgent need to meet the rising demand for mental health services with adequate and accessible resources.

However, campus mental health services do not currently have adequate means to assess the evolving nature of demand or needs. While periodic surveys of students’ mental health provides some barometer of mental health incidence, in terms of medication use, daily lifestyle, suicidal thoughts, depression symptoms, as well as potentially contributing academic, environmental, personal, and social factors^6^, they are accurate only in snapshots, and are prone to retrospective and susceptible to biases^7^. Since it is practically and financially unsustainable to administer surveys in real-time, when administered after a while from actual incidences, such data may not capture the time-sensitive nature of mental health needs and demands. Surveys are also difficult to scale and are limited to smaller study groups. With an increasing gap in the supply of mental health resources and their growing demand, college campuses need to find alternative means to gauge and forecast the demand of counselling services in order to cater to everyone who needs them.

To overcome such limitations, researchers and practitioners have started exploring passive sources of data, which provide dense and longitudinal behavior of individuals at scale^8^. Given the ubiquity and widespread use of social media, especially among the college student demographic, social media data has also been leveraged as a “passive sensor” that can act as a complementary source of unobtrusive, real-time, and naturalistic data to infer wellbeing^9^. Social media data is low-cost, large-scale, non-intrusive to collect, and has the potential to comprehensively reveal naturalistic patterns of mood, behavior, cognition, psychological states and social milieu, both in real-time and across longitudinal time for individuals and collectives^10^. Social media language consists of an individual’s personal and social discourse about day-to-day concerns, and effectively reflects their health and psychosocial wellbeing in a variety of states and contexts^10–14^. Linguistic cues and social interactions on social media platforms have therefore, enabled researchers to study psychopathologies including depression, anxiety, stress, and loneliness^15–19^. Closely related to this research, Guntuku et al. showed how social media language distinctly associates with healthcare utilization, in terms of emergency and inpatient hospital visits^20^.

Social media plays a pivotal role in college students’ lives, and is reflective of their behavior and psychosocial wellbeing^21–25^. Social media helps the college students to draw social capital benefits^26^, to satisfy psychosocial needs^22,27^, and seek solidarity and support during both normalcy and crisis^23^. Recently, researchers have studied college student wellbeing through campus-specific discussions on Reddit, which function as online analogs of offline and geographically situated college communities^23,24,28^. Bagroy et al. measured campus-specific Mental Wellbeing Index (MWI), and found seasonal trends of mental health expressions which were higher during academic terms as compared to holidays^24^, and Saha et al. measured the efficacy of counseling recommendations following student deaths on college campuses^28^.

Although considerable research reveals the potential of social media data, its predictive ability in correspondence with *ground-truth* mental health data in colleges is yet to be validated. If validated, social media data can be appropriated to help meet the varying demands of college students’ mental healthcare at various points of the academic year both in normalcy and crisis, such as the ongoing COVID-19 pandemic. This study targets this previously unexplored problem to examine if social media expressions of college students reflect mental health service utilization by college students. We conduct our study on a Reddit dataset of the online community of a large U.S. public university, which includes 66,020 posts by 18,401 unique users. We employ transfer learning classifiers to identify the language indicative of symptomatic mental health expressions of depression, anxiety, stress, suicidal ideation, and psychosis. We build seasonal auto-regressive time series models using the monthly number of mental health consultations (ground-truth) in the same university campus. We also examine the social media language using unsupervised language modeling and psycholinguistic characterization to explain the predictive ability of this data in terms of how it captures mental health concerns and challenges faced by college students. Our study bears positive implications on resource planning and management of on-campus mental health services. The ability to forecast demand of mental health services can enable on-campus health centers to plan better and cater to the students’ needs during critical times.

## Results

### Inferring symptomatic mental health expressions on social media

**Figure 1:**
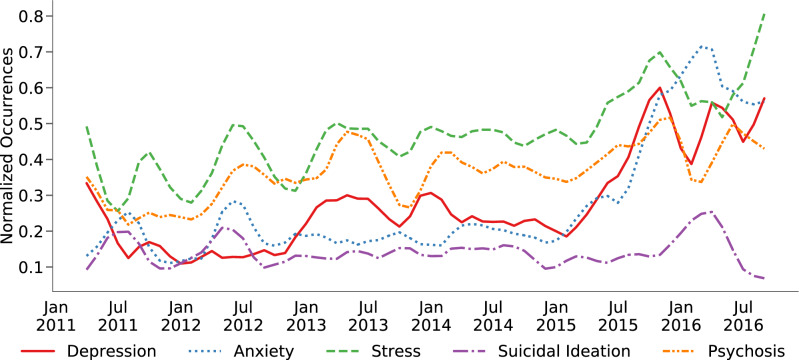
Temporal evolution of the normalized prevalence of social media expressions indicative of mental health symptomatic outcomes on the college subreddit.

We employed transfer learning classifiers to identify language indicative of depression, anxiety, stress, suicidal ideation, and psychosis on the university’s subreddit posts. Out of the 66,020 total posts in 2011–2016, 23.49% were related to depression, 21.62% to anxiety, 42.23% to stress, 14.19% to suicidal ideation, and 31.94% to psychosis. We aggregated the monthly averages of each type, and Fig. 1 shows the temporal evolution of these posts. Interestingly, greatest mental health expressions occur in April, July, and November, which also coincide with the periods before examinations for the university in consideration, as well as that for most U.S. colleges that follow three-semester cycle in an academic term (spring, summer, and fall semesters). On the other hand, the mental health expressions are observed to be lowest in December, February, and October, which coincide with winter, spring, and fall holidays respectively.

### Associating social media expressions and on-campus mental health consultations

Next, we examined if inferring the symptomatic mental health expressions bears relevance to the ground-truth data of on-campus mental health consultations. Figure 1 also shows some form of trend and seasonality in the occurrence of symptomatic mental health expressions. A Dicky-Fuller test revealed that these time series are not stationary ($p>0.05$). Therefore, for each time series, we conducted trend and seasonality decomposition, and applied moving window based trend and seasonality removal to obtain transformed residual time series that passed the stationarity test ($p<0.05$).

**Figure 2:**
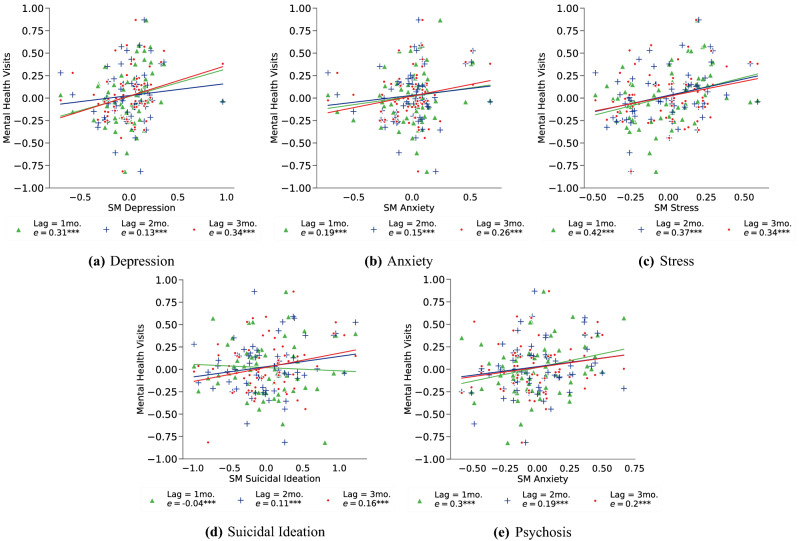
Lagged cross-correlation between the trend-seasonality removed residuals of on-campus mental health consultations (visits) and mental health symptomatic expressions on social media (SM).

We conducted similar time series decomposition on our ground-truth data. Then, we obtained the cross-correlation between the residual time series of social media mental health expressions and ground-truth data of mental health visits. We built linear regression models at various lags by controlling for base-rates of the previous month’s number of mental health consultations and the prevalence of mental health expressions on social media. A lag of *n* months indicates a comparison where the social media data is shifted by *n* months behind the ground-truth data. A higher standardized coefficient would explain a greater predictive ability of the social media expressions towards the ground-truth data. Next, Fig. 2 plots and reports the cross-correlations for each of the mental health symptomatic expressions. Except for suicidal ideation, we find a positive correlation coefficient for all other measures. We find the greatest coefficient for stress (mean *e* = 0.38), followed by depression (mean *e* = 0.26), psychosis (mean *e* = 0.23), and anxiety (mean *e* = 0.20); all with statistical significance ($p<0.05$). These correlations suggest that social media data can be potentially effective to predict on-campus mental health consultations.

### Predicting on-campus mental health consultations

Now, we predict on-campus mental health consultations using seasonal auto-regressive integrated moving average (SARIMA) time series modeling. Table 1 shows the predictive performance of the two models, $M_0$ and $M_1$−$M_0$ is the *baseline model* which uses only the time series of on-campus mental health consultation data, and $M_1$ combines the time series of on-campus mental health consultation data and mental health expressions captured from the college subreddit.

**Table 1:** Seasonal Auto Regressive Integrated Moving Average (SARIMA) models of predicting normalized number of mental health consultations in the test dataset (year 2016 data) (*$p<0.01$, **$p<0.001$, ***$p<0.0001$).

Model	Pearson’s r	MAE	SMAPE
Model $M_0$	0.76***	2.51	22.64
Model $M_1$	0.86***	1.96	13.30

Dependent overlapping correlation: *t* = − 2.07**.

**Figure 3:**
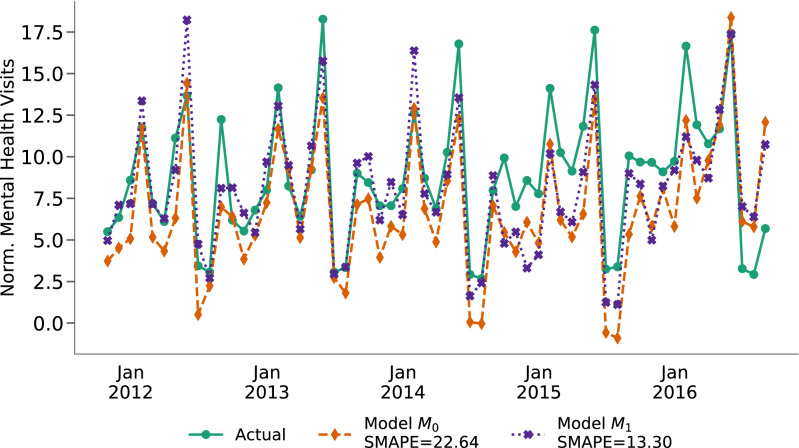
SARIMA models to predict the number of on-campus mental health consultations (visits).

We find that $M_1$ shows 13.16% better correlation and 41.25% lower error than $M_0$. A dependent overlapping correlation between the two model predictions shows a statistical significance (*t* = − 2.07, $p<0.01$). Figure 3 shows the model predictions in comparison to the actual values. Drawing on permutation test approaches^29,30^, we permuted (randomized) the predictions of mental health consultations. 1000 such permutations of randomized predictions show a Pearson’s *r* = 0.09 and SMAPE = 32.40 at average, and a probability of 0 of better performance than either of $M_0$ or $M_1$. This rejects the null hypothesis that any prediction improvement is by chance. Overall, our results reveal that combining baseline model ($M_0$) with social media based inferences of symptomatic mental health outcomes (in $M_1$) is an effective means to predict on-campus mental health consultations.

### Examining how social media language explains mental health consultations

Finally, we illuminate the characteristics of social media language that corresponds with our ground-truth. For this, we separated the social media data of the months that showed high mental health consultations (Hi-MHC) and those that showed low mental health consultations (Lo-MHC) on a median split. We conducted two types of language analysis, which we describe below.

#### Analyzing linguistic cues

First, we employed an unsupervised language modeling technique called Sparse Additive Generative Model (SAGE). Table 2 shows the most salient keywords distinctly used in Hi-MHC and Lo-MHC months. We find that the Hi-MHC months show greater prevalence of keywords related to academics and examination, such as, “finals”, “hours semester”, “summer classes”, “textbooks”, etc, and keywords related to disciplines such as, “cs majors”, “geology”, and “psychology”, e.g., “I need urgent help. I’m about to get kicked out of my CS major. I need a 2.65 entry level GPA to advance. I made an A—in 312 and a C—in data structures, so my CS gpa at 2.66.” Hi-MHC also show keywords related to “commencement” and “graduation”, which could associate with the stress during post-college transition period of students^31^, for instance, “When my met my advisor to apply for graduation he told me that I needed a BF certificate to count as my minor, I wish I knew this before.”

**Table 2:**
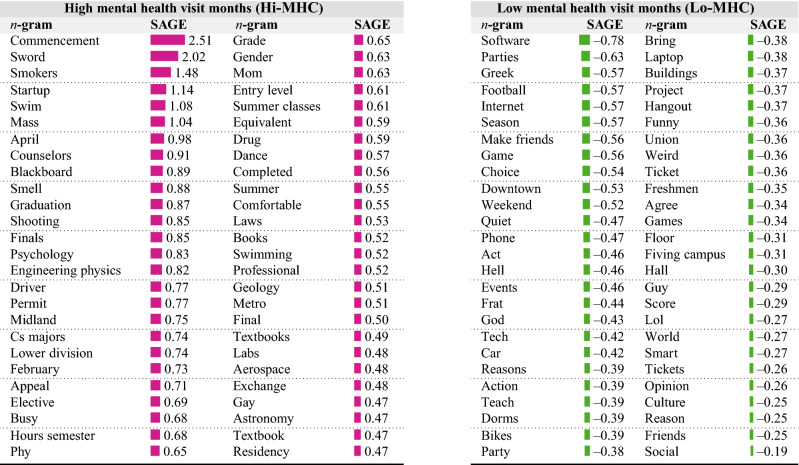
Top salient *n*-grams (*n* = 1, 2, 3) distinguishing months of high and low mental health visits as per SAGE^49^.

Bar lengths and color indicate magnitude and sign of SAGE value; **pink** bars (positive SAGE) indicate a saliency in Hi-MHC months whereas **green** bars (negative SAGE) indicate a saliency in Lo-MHC months.

In contrast, Lo-MHC months show a greater prevalence of keywords related to events, such as “parties”, “football”, “events”, “hangout”, and “social”, such as “The parties were pretty lame and we were bored at one. My friends and I stole some beers and broke into a pool only to get nearly arrested!” Likewise, Lo-MHC also show keywords related to people and friends. Other forms of social gatherings such as “game” and “football”, and accommodations such as “frat” and “dorm” occur saliently in the Lo-MHC months, e.g., “I’m a freshman, currently pledging a frat! I like partying, programming, drinking, playing, lifting weights, and mindlessly scrolling social media for hours!.”

#### Psycholinguistic characterization

We next discuss the results from our psycholinguistic characterization. First, we extracted the normalized occurrences of the 50 psycholinguistic categories as per LIWC^50^. Then, for each category, we conducted an independent sample *t*-test between the occurrences in Hi-MHC and Lo-MHC months followed by a Benjamini-Hochberg-Yekutieli False Discovery Rate (FDR) correction. We present the results in Table 3.

**Table 3:**
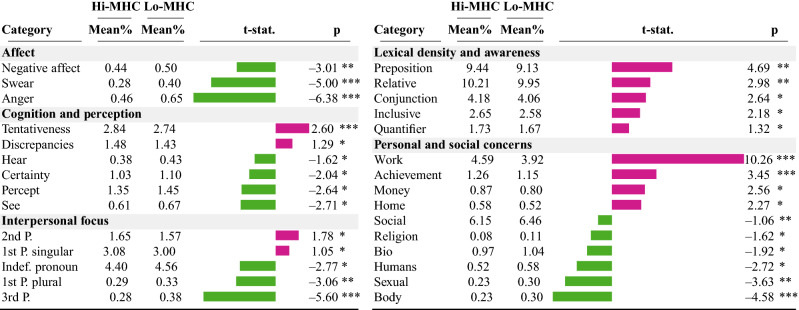
Comparing psycholinguistic attributes across clusters, with mean normalized percentage occurrence in Hi-MHC and Lo-MHC months and *t*-test statistic.

Statistical significance reported after Benjamini-Hochberg- Yekutieli False Discovery Rate correction ($$***$$ $$p<.001$$, $$**$$ .001< *p* $$<.01$$, $$*$$ .01< *p* <.05). Bar length and color indicates magnitude and sign of *t*-statistics. **Pink** bars (positive *t*) indicate greater prevalence in Hi-MHC months and **green** bars (negative *t*) indicate a a greater prevalence in Lo-MHC months.

##### Affective and cognitive attributes

Affective and cognitive attributes are indicative of an individual’s disclosure and expressiveness in social media language. Among affective attributes, we find that Lo-MHC months show greater prevalence of affective categories, including *anger*, *negative affect*, and *swear*. Although all of these categories bear a negative connotation, their greater occurrence reflects greater expressiveness, which is known to be a positive wellbeing indicator^32^. This might associate with people venting out more often about their campus life, such as in, “Now I have even more reason to not live here next year. Fuck this place!” Among cognitive attributes, we find that the Hi-MHC months show a greater prevalence of *tentativeness* and *discrepancies*, which indicate an individual’s insecurity and low degree of immediacy about the situation^50,33^. In contrast, the Lo-MHC months show a greater prevalence of *certainty*, *percept*, *hear*, and *see*, e.g., “If you want to save your bandwidth, go to a computer lab, and watch youtube/listen to grooveshark/watch netflix all day long.” The greater use of these category of language has been associated with an individual’s better cognitive functioning and mental health^34^.

##### Linguistic style attributes

We first examine pronoun usage; pronouns are markers of social attention and connectedness^35^. We find that Hi-MHC months show a greater prevalence of *first person singular* and *second person pronouns* — these could be indicative of heightened self-attentional focus, first-hand accounts of personal events, narrative, and conversational language^34^, for example, “I added psychology in my second year. I have learned that this is a very rigorous path to take, a huge commitment, and that you may need to take an extra year to complete. I must have taken at least 15 semester hours for every semester I spent here, peaking at 21 h last semester.”, where an individual describes the challenges of their college journey. In contrast, Lo-MHC months show a greater prevalence of first person plural pronoun which associates with narrating as a collective identity^23,36^, such as in “There are plenty of ways to socialize here, as we have several student organizations.”. We also see a greater use of several function words including *preposition*, *conjunction*, *relative*, and *inclusive* in the Hi-MHC months, which are known to relate with personal narrative writing style^36^.

##### Personal and social concerns

Among personal and social attributes, we find that Hi-MHC show a greater usage of *work* and *achievement* keywords, which could associate with discussions on career and self-actualization, and a greater use of *money* may associate with students discussing financial concerns, e.g., “Is it possible to consolidate jobs, save money and improve level of service? What would be an implementation to achieve this?”. Hi-MHC also show a greater use of keywords related to *home*, which could include challenges with roommate, e.g., “I got stuck on a top floor between a bad roommate and an old, tiny room, such a terrible year.” In contrast, Lo-MHC months show a greater use of social words, such as in “I have a lot of free time and realized I really don’t have a lot of friends. I’ve always been a social person, but it’s been hard to make friends at this time of the year, since classes and clubs and everything are ending. How do you recommend I meet some new peeps?”.

## Discussion

### Principal findings

This study showed that social media interactions of college students can help predict ground-truth data of on-campus mental health consultations. We adopted machine learning approaches to infer mental health expressions on a university’s Reddit community, and then incorporated the model outcomes in time series forecasting models of the normalized number of on-campus mental health consultations. First, we found that (online) mental health expressions of college students correlate with (offline) mental health service utilization on college campus. Second, we found that the SARIMA model of forecasting on-campus mental health consultations accounting for social media data could predict the ground truth within 10.65% of error, which was also 38% lower error than models that did not include social media data. Finally, we conducted a deeper dive into the language of social media posts by comparing the data of months with high and low mental health visits using psycholinguistic characterization and an unsupervised language modeling called SAGE. We found that the months of high mental health visits tend to show a greater prevalence of words related to academics, academic examinations, career, and psycholinguistic attributes indicative of worse mental wellbeing, whereas the months of lower mental health visits show a greater prevalence of words related to social, partying, leisure, and psycholinguistic attributes indicative of better mental health. Together, social media data bears the ability to capture the language and social interaction of college students, and therefore can function as a “verbal sensor” to assess mental health needs and demands of college students.

### Methodological and practical implications

This work establishes the construct validity of computational assessments of mental health from social media data. This data can therefore serve as an unobtrusive and passive lens to gauge offline critical measures that are otherwise challenging to predict, including other forms of mental health service utilization, such as uptake of peer support interventions, should that type of data be accessible or easily gathered. Our study also demonstrated the face validity of this data, where it revealed discussions and concerns related to local, contextual, and contemporary events of interest, for example, during certain political event on gun laws in U.S., a student posted, “It’s fucking nonsensical to carry a pistol around campus despite a handgun license!” Likewise, following a student death on the campus, students felt stressed and anxious about the event, e.g., “It is so depressing! Seems like he jumped out wanting to die.” This construct and face validity showcases promise that, since our machine learning models were built on considerable amounts of social media data, we believe they would be applicable across time periods of various lengths, capturing the ebbs and flows of a typical academic year, such as expectedly stressful periods as well as those when students typically recuperate and rejuvenate. Nevertheless, we note that due to the underlying sensitive nature of the mental health consultation data and practical challenges in gathering and gaining access to it, we had to rely on data from a single university. Consequently, we cannot claim generalizability at this stage. Still, this paper provides a first feasibility study of validity of social media data that can be extended in future research, spanning different universities, contexts, and datasets.

Next, this work provides empirical evidence that can help to move toward constructing practical applications of on-campus mental health assessments using passive and unobtrusive data sources. Recent research has highlighted the extent to which stakeholders—including campus stakeholders and more generally, clinicians—value the potential of these technologies, such as in the form of proactive mental health assessment tools^37,38^. This work established the construct and face validity of these assessments, as described above, and therefore, can guide building tools and dashboards that proactively assess the mental health of college students from online social chatter. Although not ready for real-world use immediately, we foresee two applications that our work could inspire.

The first application sounds ways to assess campus pulse or campus morale—timely, contextual information regarding the mental wellbeing of students. These can be in the form of interfaces, visualizations, and systems^37^ which help college stakeholders, including administrators, policymakers, and wellbeing councils, to gauge the needs of the students and accordingly ensure that adequate resources are available and measures are taken to meet the demands of mental health related services. Because our approach can yield assessments over time, the models can further be used to capture the ebbs and highs of mental wellbeing as well as its temporally-varying and evolving characteristics, such as during a typical academic year. Since our methods were predictive of mental health consultations on campus, these assessments could also be used to understand the impacts of academic events like examinations, regulations and policy decisions in campus life.

A second application of this work could center around facilitating improved preparedness in campus in case of an emergency or crisis, and assessing mental resilience of the student body in response to adverse events that affect mental well-being of student (e.g., shooting incident on campus^23^, an infectious disease outbreak like COVID-19^39^, and so on. Speaking more specfically, such preparedness may mean managing/increasing the allocation of resources in the student health clinics on campus in the form of available clinicians or consulting hours, amplifying avenues for seeking alternative sources of mental health help, such as peer support, peer counseling, or crisis rehabilitation services, or even organizing awareness and educational initiatives/campaigns that encourage students to seek help and care more proactively. In essence, with our models, decision-making and resource allocation around college student mental health could be made more evidence-based.

### Limitations and future directions

Our study has limitations, some of which also suggest novel and impactful future directions. We cannot claim clinical validity to our assessments, and building upon prior work^40^ is a direction to evaluate in the future. The findings of our study is limited to one college campus and a single form of ground truth data (on-campus mental health consultations). However, our computational approaches can be translated and adapted on other college campuses and for other wellbeing measures. We note that social media data suffer from limitations of sparsity and self-selection, i.e., this data only allows us to observe those who use and choose to post on social media. Therefore, the utility of these approaches are bounded by how active and generally engaged the social media discussion board and students of a college campus are, although we expect our methods to be applicable to comparably sized institutions and with similar demographic makeup as the one studied in this paper. Prior work noted that Reddit communities with at least 500 subscribers are somewhat representative of the campus population^23,24^. Future research can thus expand the models developed here to varied university settings with active social media presence, such as a rural or suburban institution, or a liberal arts college, to test generalizability and robustness of the construct validity findings explored in this research. Further, as in the case of any large data source, Reddit data is not immune to noise. Despite moderation strategies, this data can contain discussions irrelevant to personal and campus lives of students (e.g., advertisements, promotions, etc.), and members who do not belong to the college communities—these need to be accounted for when considering practical implementations of computational and data-centric assessments. Future work can also validate mental wellbeing assessments from other social media streams that allow longitudinal posting, instantaneous interactions, and private socializations such as Facebook, Twitter, or Snapchat, which can provide complementary information about individual and collective mental health on college campuses. Finally, replicating and reproducing the validity results from this work to other types of mental health service utilization data would bolster confidence in the application of our methods to real world settings.

## Methods

### Ground-truth data of on-campus mental health service utilization

This research builds upon health center data stemming from a large public university in the southern U.S. with an enrollment of over 50,000 students. Our ground-truth dataset comprises the count of monthly health center visits by students at the same university. The visits are classified into two types: visits related to mental health issues, and those unrelated to mental health issues. This data spans a period of 84 months: September 2009 to August 2016.

For the purposes of our study, we normalized the monthly measure of mental health consultations as the percentage of enrolled students who sought mental health service in the same month. Such a normalization facilitates two goals—(1) minimalization of confounding outliers and distortion due to total number of enrolled students; and (2) preservation of the privacy of the university and the students of the university whose data is being studied.

### Social media data

We focus on the social media data pertaining to the college students from the same university. For this purpose, we used data from Reddit. Reddit is a popular social media platform among the age group between 18 and 29 years: Pew Research found that 65% of Reddit users are young adults^41^. This age group aligns with the typical college student demographic. Reddit facilitates focused conversations through “subreddits” that comprise of members interested in a specific topic. Many colleges have a dedicated subreddit community, which provides a common portal for the students on a campus to share and discuss about a variety of issues related to their personal, social, and academic life^23,24^. Reddit is suitable data source for the study as it allows us to isolate posts from students from a particular college campus. Reddit, by design, facilitates candid disclosures by allowing pseudonymous and throwaway accounts, and community-driven moderation to maintain authenticity of members and civility and relevance of discussions^42–44^. In the case of college subreddits, the members often need to provide proof of their authenticity status to the moderators before participating in the discussions. While the subreddits also remain open to the alumni and staff, typically, only active students engage the most in ongoing discussion threads. Prior work has also leveraged Reddit data to study college students^23,24,28,45^. We obtained the data from the subreddit corresponding to the same university under study, using the BigQuery API which hosts Reddit data archives^23,24^. This archive included 66,020 posts by 18,401 unique users averaging at 33 posts per day between May 2011 and August 2016. The rest of the paper studies this period as this overlaps with our ground-truth data availability as well.

### Modeling approach

Our primary objective concerned examining if the online college community data is reflective of on-campus mental health service consultations. We identified the language indicative of symptomatic mental health outcomes from these social media posts. Then, we conducted time-series modeling to predict the mental health visits. We evaluated if including information gathered from social media data improved the predictions.

#### Measuring symptomatic mental health expressions on social media data

We quantified mental health related expression in Reddit posts using machine learning classifiers identifying the language indicative of symptomatic mental health expressions of depression, anxiety, stress, suicidal ideation, and psychosis. We adopted the approach presented in^17^. Essentially, these classifiers are built using transfer learning methodologies, i.e., transferring a classifier trained on a different labeled dataset. These classifiers are *n*-gram (*n* = 1,2,3) based binary SVM models where the positive class of the training datasets come from appropriate subreddits, i.e., *r/depression* for depression, *r/anxiety* for anxiety, *r/stress* for stress, *r/SuicideWatch* for suicidal ideation, and *r/psychosis* for psychosis, and the negative class of training data comes from non-mental health content on Reddit—a collated sample of 20M posts, gathered from 20 subreddits from Reddit’s home page such as *r/AskReddit*, *r/aww*, *r/movies*, etc. These classifiers perform at a high accuracy of approximately 0.90 on test data^17^. We used the classifiers to label each post in our Reddit dataset with binary (0 or 1) labels of each symptomatic mental health expression.

#### Predicting mental health service utilization

To predict monthly mental health consultations, we adopted a time series modeling approach. We used seasonal auto regressive integrated moving average techniques (SARIMA)—a standard time series forecasting method based on past behavior accounting for seasonality^46^. SARIMA incorporates seasonality in addition to auto regressive integrated moving average techniques (ARIMA)^47^, and is suitable in time series with seasonality (e.g., in our case there is known seasonality in academic cycles). We draw on *k*-fold (*k* = 10) cross-validation approach to predict and evaluate our modeling approaches. We first set aside the data from the first year of our dataset (2011) as the default training set so that the models could learn from the same baseline historical data. Then, we obtained various combinations of tenfolds, i.e., 90% of the remaining data was used to build a model that predicted the monthly mental health consultations on the remaining 10% data, and we iterated on various combinations to predict the entire dataset. As our work primarily targets the efficacy of social media data in understanding mental health service utilization, we built two kinds of models, as listed below.

- Model $M_0$ is trained using only the time series of on-campus mental health consultation data. This model can be considered to be the one used in most in-practice purposes, or as our *baseline model*.
- Model $M_1$ is trained using the time series of on-campus mental health consultation data, in conjunction with the time series of monthly aggregated mental health discussions in social media. For this, we calculate the monthly average of posts relating to depression, anxiety, stress, and suicidal ideation as identified by our classifiers.

We used the above trained models to separately predict the number of monthly consultations in test data. We pooled all the predictions together to compare against the actual values and compute the Pearson correlation coefficient (*r*), where higher values directly associate with better performance. We also measured the prediction error between the actual and predicted data as mean absolute error (MAE) and symmetric mean absolute percent error (SMAPE). MAE calculates the arithmetic average of the absolute errors ($|y_i-x_i|$) where $y_i$ and $x_i$ are the predicted and actual values respectively, and SMAPE calculates percentage of relative errors ($|y_i-x_i|/[(|y_i|+|x_i|)/2]$), and is bounded between 0 and 100. For both error measures, lower values indicate lower error and better predictive performance.

While comparing $M_0$ and $M_1$, if $M_1$ shows comparatively better predictive performance than $M_0$, we would conclude that using social media data contributes to better predict on-campus monthly mental health consultations. To measure statistical significance in prediction differences between $M_0$ and $M_1$, we conducted *t*-tests using the dependent overlapping correlation method, which controls for comparing against a common variable of interest (here, the ground-truth number of monthly on-campus mental health consultations)^48^.

### Analyzing the social media language of mental health

Finally, we interpreted how social media language associates with on-campus mental health consultations. We obtained the months of high and low number of mental health visits—we adopted a median split on the normalized number of visits in a month. Then, we examined the distinction of these periods as per social media language. This examination would help establish the face validity of the social media language in correspondence to the ground-truth. We conducted two analyses:

First, we adopted an unsupervised language modeling technique called the Sparse Additive Generative Model (SAGE)^49^. Given two documents, SAGE finds the keywords that distinguish the documents by comparing the parameters of two logistically parameterized multinomial models using a self-tuned regularization parameter that controls the tradeoff between frequent and rare terms. We aimed to obtain keywords that would relate with the key concerns faced by college students that lead to heightened mental health concerns.

Second, we conducted a psycholinguistic analysis. For this, we used the well-validated psycholinguistic lexicon, Linguistic Inquiry and Word Count (LIWC)^50^. LIWC characterizes social media language in 50 psycholinguistic attributes ranging across affect, cognition and perception, interpersonal focus, temporal references, lexical density and awareness, and personal and social concerns. This analysis would help to contextualize the social media language of college students in the literature on mental health and therefore explain the predictive ability of social media language.
